# Self-Reported Changes in Risk Behaviours of Cardiovascular Diseases among School Adolescents in Nepal: Application of an Integrated Experiential Learning Approach

**DOI:** 10.5334/gh.818

**Published:** 2020-05-21

**Authors:** Rita Thapa, Raj Kumar Subedi, Gorakh Regmi, Radhika Thapaliya, Abhinav Vaidya, Benu B. Karki

**Affiliations:** 1 Bhaskar-Tejshree Memorial Foundation, Kathmandu, NP; 2 Ministry of Health and Population, NP; 3 Department of Community Medicine, Kathmandu Medical College, NP

**Keywords:** adolescents, cardiovascular diseases, experiential learning, risk behaviour, Nepal

## Abstract

Cardiovascular diseases (CVDs) account for the largest proportion of all deaths in Nepal (30%). Studies report that CVDs often begin with modifiable lifestyle risk behaviours established during adolescence which manifest later. This study aimed to measure changes in the five mortality-associated CVD risk behaviours (i.e., consumption of tobacco, alcohol, and junk food, physical inactivity, and stress among school adolescents) using an integrative intervention with the experiential learning approach. The study was carried out for 24 weeks (25 credit hours) among 4,225 students from grades 8 to 10 in community schools in seven provinces in Nepal. Pre- and post-intervention in-class self-reported surveys were conducted for assessing change in the students’ aggregate risk behaviours. These percent changes were assessed through bivariate analysis. Key-informant interviews of teachers were conducted to assess their perceptions of the effectiveness of the intervention. At 24 weeks, the percentages of students reporting consumption of cigarettes and smokeless tobacco declined by 25% and 28% respectively, consumption of alcohol declined by 29%, consumption of instant noodles by 11%, and consumption of coke by 43%. The proportion of students reporting ‘going to school by foot every day’ increased by 11%, and those ‘exercising until they sweat’ increased by 29%. The percentage of students who reported feeling that their ‘life has been running as desired’ increased by 16%. Key-informant interviews of the teachers revealed that the intervention had contributed to improved motivation, knowledge, and attitude among students towards mitigating the risk behaviours. These interviews also recommended continuation of the intervention. The sample in this study has shown positive changes in school adolescents’ self-reported aggregate CVD risk behaviours using the experiential learning approach. However, further research should be conducted to explore the sustainability and scaling of these learning modules through the existing non-communicable disease (NCD) school curriculum activities in Nepal.

## Introduction

Globally, more than 80% of all premature deaths are attributed to four non-communicable diseases (NCDs), namely cardiovascular diseases (CVDs), cancers, respiratory diseases, and diabetes [1]. In Nepal, CVDs account for 30% of all deaths [2], thus the leading causes of deaths. Several studies have shown that the process of CVDs begins much earlier, often with lifestyle risk behaviours established during adolescence, manifesting mostly during midlife [3,4]. Studies have identified the consumption of tobacco, alcohol, and junk food, physical inactivity, and mental stress as proven modifiable CVD risk behaviours [5]. At the same time, the World Health Organization (WHO) acknowledges that the consumption of tobacco, alcohol, and junk food, and physical inactivity are mortality-associated modifiable CVD risk behaviours [6]. Such risk behaviours are persistently high among Nepalese adolescents, the largest segment of the total population (23%) [7]. They all contribute to increased possibility of premature CVD-NCD deaths [8].

The 2015 Nepal global school-based student health survey (GSHS) reports that the prevalence of CVD risk behaviours among school adolescents of 13–17 years of age is 8% tobacco smoking, 5.2% alcohol drinking, a mere 15% physically active on all 7 days, 13.7% seriously contemplating suicide, 32% consuming unhealthy carbonated soft drinks one or more times a day, and 6.1% overweight [9]. Similarly, the 2015 baseline study conducted under the aegis of the Bhaskar Initiative for School Heart-Health Empowerment Studies (BISHES) project among selected school adolescents of the Kathmandu Valley in 2015 shows that school adolescents are not only faced with the CVD behavioural risks namely smoking, consumption of alcohol, eating junk food, and physical inactivity, but also with mental stress that overarches the other risks as expressed by both teachers and students. Eating junk food was common, often because of a lack of other options. Public schools, in particular, lacked infrastructure and resources to provide proper meals to students and engage them in physical activities.

Moreover, the BISHES baseline study’s analysis of the existing strengths, weaknesses, opportunities, and threats (SWOT) helped to gain deep understanding of the issue and was instrumental in designing learning materials, especially the risk-based BISHES action (BAT) intervention, using experiential learning approach, as flagged by students and teachers alike [10].

Although most of the students correctly identified the CVD behavioural risks like smoking, consumption of alcohol, consumption of fatty foods, and physical inactivity, they felt unable to put their knowledge into practice. They expressed the need for some practical learning that could enable them to bring their knowledge into practice.

Furthermore, the BISHES-commissioned review of the existing NCD health education curriculum of government schools reported that, although some contents on NCD are present in the existing curriculum, they are not sufficient [11]. The subject, Health and Physical Education, is compulsory in grade 8, while it is optional in grades 9–10. Similarly, the subject, Health, Population, and Environment, is compulsory in grades 9–10. However, practical experiential learning, an important felt need of both students and teachers, is missing.

In response to the above felt needs, BISHES developed integrated experiential learning modules on the five mortality associated CVD risks behaviours targeting school adolescents. Each module is delivered using game-based experimental learning, which has been demonstrated as an effective experiential learning method for more than two decades [12]. BAT’s experiential learning modules are adapted to the theoretical framework of Kolb’s experiential learning model [13]. They are delivered using the 5-Step Learning Cycle, which has been found effective in transmitting knowledge from direct experience [14].

Pilot testing of the BAT in three districts of Nepal viz. Illam, Tanahu, and Lalitpur demonstrated enthusiastic participation from both students and teachers, describing it as ‘learning from simplicity to complexity with fun’.

## Objective

This study aimed to measure the changes in the five modifiable CVD risk behaviours, namely consumption of tobacco, alcohol, and junk food, physical inactivity, and stress, among school adolescents before and after 25 credit hours of continued BAT experiential learning intervention.

### BISHES Intervention Action Tool (BAT)

In view of the felt need for a practical learning approach expressed by both students and teachers, and using the existing empirical evidence [13,14], the project started developing a risk-based integrative intervention with the experiential learning approach. The BAT developed in collaboration and partnership of several relevant sectors: the Ministry of Health and Population (MoHP), Ministry of Education (MoE), schoolteachers, students, local elected representatives, and public health consultants, CVD and WHO experts. The process lasted for more than two years, involving a series of activities, which are described below.

The first phase activities included an in-depth analysis of the situation and literature review with particular emphasis on the search of proven interventions on CVD risk behaviour reduction, including the learning methods that could help students learn from direct experience. The second phase activities led to weaving the above-mentioned researched contents and learning methods into template modules, identifying learning objectives for each module appropriate to grades 8–10, relevant experiments, lesson plans, and assessment forms, including teachers’ guidelines. These contents were put together to design interventions in each of the five modifiable CVD risk behaviours, i.e., consumption of tobacco, alcohol and junk food, physical inactivity, and stress. Intervention in each of these risk behaviours was designed into a template of experiential learning module by adapting to the five-step learning cycle (LC) [14]. Accordingly, a set of appropriate experiments for each risk behaviour was identified through research and entrenched into the modules concerned. Each module was intended to enable students to learn the harmful effects of each risk from direct experience. These were carried out through a cascade of mini and major workshops, drawing technical inputs from teachers, students, MoHP, MoE, and public health and CVD experts.

**Figure 1:**
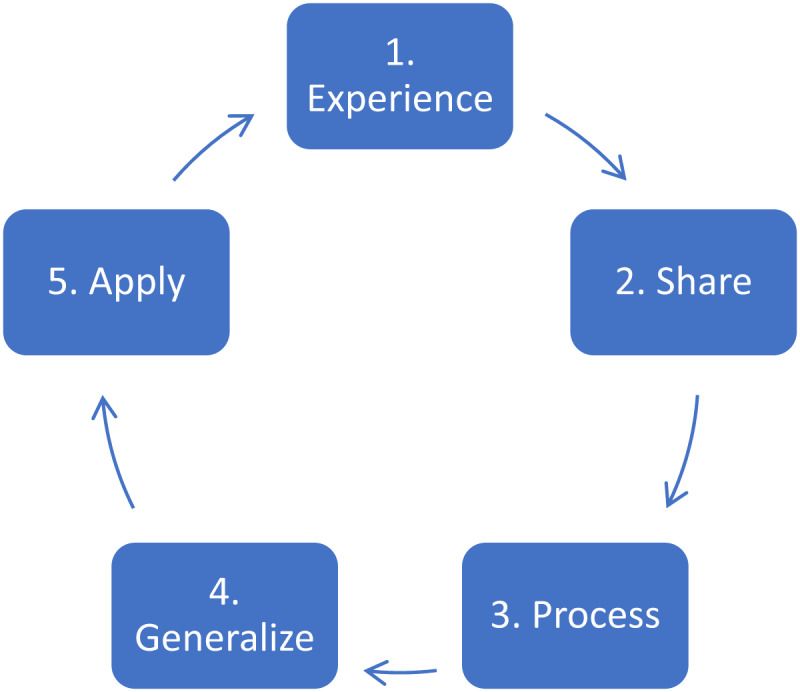
Five-step experiential learning cycle [14].

The protocol required each module to start with experiments, sequentially followed by the remaining four steps of the LC, as shown in Figure 1.

This method enabled the students to learn about the CVD risks from direct experience. For instance, in the tobacco module, we used cigarettes, plastic bottles, and tissue paper as materials to create a ‘smoking bottle’ (analogous to smoking lungs), and, by pumping out smoke from the ‘smoking bottle’, students could directly observe nicotine tar deposits extracted from cigarette smoke [15]. Likewise, students doing the ‘running with straw’ experiment experienced the simulation of difficulty in breathing by chronic smokers, caused by nicotine-tar deposits narrowing and hardening their elastic breathing passages [16]. Similarly, students performed the ‘heart pump’ experiment, experiencing how a nicotine-stimulated faster heart rate makes the heart overworked and tired, eventually stopping it [17]. In case of the alcohol module, students, by playing the ‘meeting a friend unexpectedly’ based on ‘alcohol ball’ [18], which required ‘spinning around with closed eyes’, experienced the simulation of the physical and mental effects of alcohol consumption. Similarly, students, by boiling coke, could directly see and feel the sticky dark brown residue—the phosphoric acid used to mask the excessive sugary taste of coke—as in many other soda drinks [19]. Examples of other direct learning experiments that were conducted were ‘mirror image’ [20] for physical activity and ‘laugh-o-logy’ [21] and ‘Anapana’ (mindfulness of breathing) [22] for stress.

The third stage entailed developing a procedure for the training of facilitators and trainers (ToT), based on the protocol of the five experiential learning modules. Guidelines were also prepared on the hands-on equipment and required supplies, including information and monitoring methods. Following this, the draft experiential learning modules, including the ToT guidelines, were pre-tested in a real classroom setting on 57 teachers and 185 students from 18 schools of the three provinces. The pre-tests were conducted against a few criteria: if the experiential learning module could capture students’ attention; if teachers could succeed in conducting practical experiments as required by the modules; if the learning objectives of each module could be achieved; and if the modules could be completed within the set class time limit of 45 minutes.

The fourth phase activities included synthesis, analysis, and incorporation of valid feedback collected during the pre-tests into the corresponding modules. Finally, the BISHES intervention action tool, thus developed, consisted of an integrative package of five experiential learning modules, one in each of the aforementioned five modifiable CVD risk behaviours. In 2017, BAT was shared at a high-level dissemination seminar involving CVD experts, representatives from Ministry of Health and Popultion (MoHP), Ministry of Education (MoE), World Health Organization (WHO), relevant Non-Government Organizations (NGOs), and the media. While the seminar provided constructive feedback, it recommended the application of BAT in schools across the country. Subsequently, BAT was accredited by the National Health Education Information and Communication Center (NHEICC)—an apex body for conducting, accrediting, and regulating health education activities in the country.

Importantly, the MoE also granted approval for applying the BAT intervention in schools countrywide in collaboration with local authorities.

## Methods

### Study design and setting

#### Criteria for sample selection

The schools were selected purposively. The criteria used included: public schools from each province; the schools suggested by the district education office and/or by local body representatives such as mayors; students of grades 8, 9, and 10 as they are the high-risk adolescent groups for initiation of CVD risk behaviours; absence of ongoing NCD intervention by other agencies; commitment of school authorities to assign three school teachers per school—one each for grades 8, 9, and 10—for taking ToT in the BAT intervention; and commitment to continue taking the intervention classes for six months.

A mixed-methods study drawing both quantitative and qualitative information was employed to measure the effects of the intervention, using experiential learning approach among 4,225 community school adolescents of grades 8, 9, and 10 from 32 public schools across the seven provinces of Nepal (Table 1).

**Table 1:** Sample size by province.

Province	Number of schools enrolled	Total number of students	% of the total sample

1	2	567	13.4
2	5	718	17.0
3	5	574	13.6
4	5	663	15.7
5	5	577	13.7
6	5	463	11.0
7	5	663	15.7
**Total**	**32**	**4225**	**100.0**

Quantitatively, in-class surveys using a self-administered rapid screening tool (RST) were applied for the assessment of students’ behaviour changes in pre- and post-intervention periods, ensuring students’ privacy and confidentiality. Percentage changes in students’ aggregate self reported behaviour after the 25 credit hours of learning were assessed in bivariate analysis.

Qualitatively, key-informant interviews of teachers were conducted to assess their perceptions of the effectiveness of the intervention, including learning, behaviour, and outcomes regarding CVD risk behaviours.

ToT workshops were designed and conducted, adhering to the principle of ‘learning by doing’. On the first day, a team of five BISHES trainers conducted one module each by involving the respective class teachers as volunteers in conducting related experiments. During the next three days, class teachers conducted classes on intervention under the close supportive guidance of the BISHES trainers. Each session ended with an evaluation of the achievement of its learning objectives within 45 minutes. Upon completion of the ToT workshops, each trained class teacher took the responsibility of conducting one module a week in her/his classes for the remaining five months. This allowed each student to have a dose of five sessions per module over a period of six months. Each participating school was provided with a kit containing the required equipment and supplies to last the duration of the intervention. After the completion of the ToT, teachers, as well as students, were provided with the BAT booklet.

In addition, the teachers were equipped with the lesson plans for the modules and registers to record and monitor the progress of the activities carried out by them during 25 credit hour learning. Joint signatures of class teachers and student representatives in the register validated the information recorded. Regular field visits and telephone calls were made by the project to ensure that the intervention went as intended. The recorded data and reports were regularly fed into the BISHES dashboards.

#### Data collection, analysis, and interpretation

A one-page self-reporting rapid screening tool (RST) comprising 18 screening questions, covering all the five CVD risk behaviours in the Nepali language, was applied to measure the dichotomised outcomes as presence or absence of individual risk behaviour. Questions relating to tobacco and alcohol were adapted to the Brief Screener for Tobacco, Alcohol & other drugs (BSTAD) and that of assessing stress were adapted to those developed by Center for Integrated Health Solutions, while those for physical activity and junk food were adapted to feedbacks from the previous BISHES baseline study. The RST was field-tested, and due parental consents were obtained prior to its administration. At the start, the students were told that it was not an academic test, and their honest responses could help in preventing the CVD-NCDs. Efforts were made to motivate students to respond as honestly and candidly while filling the RST form. Because the RST does not bear their names, their responses would remain anonymous. Utmost vigilance was kept so that students did not consult each other while filling the individual RST form. In order to avoid the Hawthorne effect in students’ responses, school teachers were not involved in administering the RST. We assume that all the respondents would answer honestly.

A student attendance sheet was used at pre-test and post-test to ensure that the same students participated at both phases of the survey.

Upon completion of the 25 credit hours of BAT intervention spread over 24 weeks, the same RST was used for collecting post-test data, which was conducted by persons other than those conducting the pre-test and the BAT training. This was to prevent the Hawthorne effect in the sense that the students might change their response at the presence of their teachers that conducted their pre-test and BAT training. Quantitative data were entered in MS-EXCEL and analysed using IBM-SPSS V.23. Differences in proportions (unpaired) were computed for the key risk factors of CVDs between the pre-test, and the post-test, and P values were computed for testing whether the differences were statistically significant (at 5% or less level of significance). This was done for each of the CVD risk behaviours as measured by the RST, hence scoring of RSTs was not necessary.

Following the post-tests, key informant interviews (KIIs) were conducted with schoolteachers to understand their perceptions of the effectiveness of the intervention. A total of 65 teachers from 22 schools participated in the KIIs. The qualitative data was analysed manually using thematic analysis and verbatim transcription. Themes for qualitative analysis included each of the learning domains of Donald Kirkpatrick’s model of training evaluation, which includes reaction, learning, behaviour, and result levels [23].

The study was approved by the Ethical Review Board, Nepal Health Research Council.

## Results

### Quantitative findings

#### Socio-demographic Characteristics

Table 2 shows the socio-demographic characteristics of the students. The majority of the students were from grade 9 (38.3%), followed by grade 8 (31.8%) and grade 10 (29.9%). Similarly, the majority of the students were female (54.2%).

**Table 2:** Distribution of students by sex and grade.

Characteristics	Frequency	Percentage

**Sex**		
Male	1,937	45.80%
Female	2,288	54.20%
**Total**	**4,225**	**100.00%**
**Grades**		
8	1,344	31.80%
9	1,618	38.30%
10	1,263	29.90%
**Total**	**4,225**	**100.00%**

#### Self-reported consumption of tobacco and alcohol by students

Table 3 shows the self-reported aggregate behaviour on the consumption of tobacco and alcohol with the percentage change between the pre-test and the post-test. It shows that the proportion of students reporting who lit a cigarette in the last 30 days preceding the survey declined by 33% (P < 0.01). Similarly, those reporting who smoked cigarette/bidi/hookah in the last 30 days declined by around one-fourth (P = 0.0005). Similarly, those reporting who consumed smokeless tobacco within the last month decreased by 28% (P = 0.0015), and those reporting who consumed any type of alcohol declined by 29% (P < 0.01).

**Table 3:** Self-reported aggregate tobacco and alcohol consumption-related behaviour.

Consumption of tobacco and alcohol (n = 4225)	Pre-test	Post-test	% decline	P Value

	Percent	Percent		

Ever lit a cigarette in the last 30 days	8.40	5.60	33.33	<0.01*
Smoked cigarette/bidi/hookah in the last 30 days	7.70	5.80	24.68	0.0005*
Consumed other smokeless tobacco in the last 30 days (yes/no)	5.00	3.60	28.00	0.0015*
Consumed any alcohol type in the last 30 days	9.60	6.80	29.17	<0.01*

* Statistically significant at P ≤ 0.05.

#### Self-reported level of stress and physical activity

Table 4 shows self-reported information of students’ aggregate behaviour level on stress and physical activity between the pre-test and the post-test. The proportion of students reporting who felt they could not control important aspects of life decreased by around 10% (P = 0.0003) and those reporting who felt increased confidence in handling problems increased by 4% (P = 0.0324). Similarly, the increase in physical exercise was also found to be statistically significant (P < 0.01).

**Table 4:** Self reported information on stress and physical activities.

Characteristics (n = 4,225)	Yes/No	Pre-test	Post-test	Change (in %)	P Value

		Percent	Percent		

Could not control important aspects of life last month	Yes	36.40	32.70	10.16	0.0003*
Confidence in handling problems last month	Yes	56.50	58.80	–4.07	0.0324*
Felt like everything was going on as planned last month	Yes	33.80	39.20	–15.98	<0.01*
Experienced difficulty in solving problems last month	Yes	33.90	31.90	5.90	0.0504
Have done Aana-paana- mindfulness of breathing	Yes	8.90	54.80	–515.73	<0.01*
Walked to school all six days	Yes	66.00	73.10	–10.76	<0.01*
Duration of physical exercise in school	None	34.00	17.80	47.65	<0.01*
	Up to half an hour	46.70	61.10	–30.84	<0.01*
	More than half an hour	14.60	18.10	–23.97	<0.01*
Played outside school till it sweated	Yes	55.10	71.30	–29.40	<0.01*

* Statistically significant at P ≤ 0.05.

#### Food and drink intake

Table 5 presents information regarding the self-reported consumption of different food items and drinks by the students at pre-test and post-test and the magnitude of change in the behaviour measured at those two points of time. There was an aggregate decline in the consumption of junk food such as instant noodles, potato chips, and processed spicy food (e.g. Kurkure), as well as unhealthy drinks, which were found to be statistically significant (P < 0.01) Similarly, the percentage of students consuming healthy food such as boiled egg, roasted maize, and roasted peanuts increased by 46% (P < 0.01). However, the aggregate difference in the proportion of students consuming beaten rice, mixed fruits, and vegetables was not found to be statistically significant (P > 0.05).

**Table 5:** Self-reported information on the consumption of healthy and unhealthy foods and drinks.

Food/drinks intake	Pre-test	Post-test	% Change	P-value

	Percent	Percent		

Instant noodles	79.60	70.50	11.43	<0.01*
Potato chips	47.60	39.40	17.23	<0.01*
Processed spicy titbits (eg Kurkure)	46.20	40.10	13.20	<0.01*
Beaten rice	59.10	57.50	2.71	0.1359
Roasted maize	52.00	60.40	–16.15	<0.01*
Roasted peanuts	39.50	57.70	–46.08	<0.01*
Boiled egg	58.00	62.60	–7.93	<0.01*
Coke	47.60	27.10	43.07	<0.01*
Fanta	37.30	22.10	40.75	<0.01*
Mixed vegetables	84.00	84.90	–1.07	0.2537
Fruits	84.2%	85.30	–1.31	0.1597

* Statistically significant at P ≤ 0.05.

#### Sex-differential in the CVD risk behaviour decline

The decline in the consumption of tobacco, alcohol, and instant noodles was found higher among female students than among male students (Table 6). Similarly, the increase in the proportion of students doing exercise until it sweated outside the school was higher for females (51.21%) than for male (16.34%) students. However, there was the same level of percentage decline among male and female students with regard to mental stress (as measured by ‘feeling like everything was going as planned’) and consumption of coke. Likewise, a same level of percentage increment was found among male and female students walking to school six days. (Table 6).

**Table 6:** Sex-wise difference in self-reported reduction in the CVD risk behaviours.

The CVD Risk Behaviurs	Relative difference in pre-test and post-test in % among sex	

	Male	Female

Ever lit a cigarette in the last 30 days	17.24	51.72*
Smoked cigarette in the last 30 days	14.18	34.38*
Consumed other smokeless tobacco	10.71	50.00*
Consumed any alcohol	11.45	47.06*
Felt like everything was going on as planned	–15.00	–16.62
Walked to school all six days	–10.58	–10.53
Played outside school till it sweated	–16.34	–51.21*
Consumed instant noodles	8.84	13.38
Consumed coke	43.69*	42.58*

*Note*: Only key variables have been used in the comparison table. Negative sign shows increment. * Statistically significant at P ≤ 0.05.

Looking at the distribution in the reduction of the key CVD risk behaviours by provinces, a decrease of more than 50% was found in the proportion of students ever lighting a cigarette in provinces 2 and 5 (Table 7). Similarly, those smoking cigarettes in the past 30 days declined by the largest percentage in province 5 (41.94%). Province 5 was also found to be the most successful in decreasing the consumption of smokeless tobacco (69.57%). Moreover, province 2 recorded the highest decline in the consumption of alcohol and also the highest reduction in mental stress (as measured by ‘feeling like everything going on as planned in the past 30 days’). Similarly, compared to other provinces, province 2 was the most successful in increasing the proportion of students who walked to school all six days a week. All provinces, except province 3, showed a more than 30% increase in the proportion of students consuming coke, whereas province 3 showed a 21.62% decrease in this respect. Province 7 showed the highest reduction in consuming coke by the students (Table 7). In sum, provinces 2, 5, and 7 were the provinces most successful in declining the five CVD risk behaviours among school adolescents.

**Table 7:** Change in key CVD risk behaviours by province.

Key CVD risk behaviours	Provinces (the relative difference between pre-test and post-test in %)						

	1	2	3	4	5	6	7

Ever lit a cigarette in the last 30 days	7.95	50.65*	39.26*	18.00	54.10*	29.82*	26.67*
Smoked cigarette in the last 30 days	4.00	34.92*	21.11	28.16*	41.94*	20.00	28.57*
Consumed other smokeless tobacco	19.39	58.21*	13.73	63.00*	69.57*	5.00	12.00
Consumed any alcohol	11.00	51.67*	28.72*	24.49*	38.10*	14.43	45.45*
Felt like everything going on as planned	–7.74	–41.60*	–14.93	–8.45	6.82	–13.33	–21.99*
Walked to school six days	–5.18	–45.05*	–6.95	–1.73	–18.27	0.55	–23.25*
Played outside school till sweated	–14.64	–28.85*	–23.48*	–26.93*	–42.95*	–15.68	–46.14*
Consumed instant noodles	–1.32	20.19*	11.06	2.03	4.10	21.32*	20.38*
Consumed coke	30.84*	40.98*	21.62	56.59*	53.76*	35.14*	67.80*

*Note*: Only key variables have been used in the comparison table. Negative sign shows increment. * Statistically significant at P ≤ 0.05.

### Qualitative Findings

KIIs with the participating schoolteachers revealed that they perceived the experiential learning programme as very useful for students, as well as schools as a whole. The questions were framed in line with Donald Kirkpatrik’s four levels of training evaluation, namely, Reaction, Learning, Behaviour, and Outcome [23].

#### View about the contents and methodology

Majority of the teachers were of the view that the contents of the training were adequate and useful. Similarly, when asked about the methodology of the course, they said that the experiential learning approach was highly pertinent to behavioural change among the students. One of the participants said, ‘I like the method because it teaches complexity from simplicity with fun; its lesson plans are very effective’.

#### A sense that it would improve students’ knowledge

All the participating teachers were of the view that the training was able to improve the existing knowledge of the students. They were of the opinion that the training had been able to impart practical skills to the students because learning was more detailed and persistent. Students are less likely to forget what they learn from experiential education. Majority of the teachers said that the students, after having learnt the five risk factors of CVDs, were also imparting the knowledge to their family members, as well as junior students. One of the teachers said, ‘This has been able to improve not only the students’ knowledge, but also my knowledge. I have learned about so many negative consequences of smoking, eating junk food, leading a sedentary lifestyle, drinking, as well as stress. This is an eyeopener for teachers as well’. This reflects that BAT has been able to arouse the school community’s interest while improving their CVD prevention knowledge.

#### Perception of behaviour change

Teachers stated that there were marked changes in the behaviour of the students after participating in the training sessions using BAT. Almost all teachers said that a notable decline was found in smoking and consumption of coke. One of the teachers said that, ‘The smoking bottle experiment is very good. It has made students internally realize the harmful effects of smoking on human lungs’.

Similarly, another teacher said, ‘The experiment of boiling coke had made the students realize how much sugar the drink contained. Students had since stopped taking the carbonated drink’. Some of the teachers even mentioned that they used to get calls from the parents of some students saying that their children had started motivating them to quit smoking and drinking. This indicates that not only BAT has been able to bring about positive changes in the risk behaviours, it has created positive ripple effects on parents as well.

#### Expectation of reduction in CVD-related deaths in communities

The teachers had perceived that the intervention would be able to contribute reducing premature deaths due to CVDs in the communities. They were of the view that students could be strong vehicles for such change.

According to them, the change could be sustained if the contents related to the prevention of CVDs could be incorporated in the school curriculum. To quote a teacher in this regard:

‘Incorporating BAT in school curriculum will be most appropriate. So far, we have educated only the students of grades 8, 9 and 10, and others have been left out. If this were not continued, the alone will not be adequate to solve the problems. If this could be included in the curriculum, the knowledge would be passed on from one batch to another. The other batches will also get an opportunity to learn and it will at last become meaningful’

One other teacher said, ‘I consider this activity a highly useful skill for life because it doesn’t cost anything, it protects us from many diseases, and even the poorest of the poor can practice it at home.’

## Discussion

As habits are entangled into the complexities of multiple factors, they do not change easily. Research, however, shows that habits can be altered when appropriate interventions are applied to a population willing for change, and, eventually, the desired change occurs when participants experientially realise its benefits [24].

A number of studies have shown that it is feasible to carry out cardio-vascular disease risk reduction programmes to a larger population through school based approaches [25,26,27]. However, the degree of effectiveness of such interventions depends on several factors. Thomas and colleagues, in their paper ‘School Based Interventions’, have pointed out that most of the school-based interventions do not sustain because the priority of schools is educating students than motivating them towards behavioural change, thus, emphasizing that if prevention programmes are incorporated as part of curricular activities, they are more likely to sustain [28].

As behaviour change is a long process going through different stages, namely Pre-contemplation, Contemplation, Determination, Action, and Maintenance [29], sustained interventions are desirable. In this regard, the key informants’ suggestion to continue the interventions for a longer duration is justifiable for ensuring sustained benefits accruing from the risk behaviour reduction.

The high level of receptivity of the intervention along with its experienced benefits, as expressed in the KIIs, may have been one of the indirect community factors associated to the positive behaviour changes [30].

Comparatively, the aggregate self-reported CVD risk behaviours at post-test of this study are lower compared to that of the Nepal 2015 global school-based survey (GSHS) and that of the 2016 BISHES baseline survey.

Table 5 shows that female students comprising 54 % of the total sample outnumbered the male students, and they show more statistically significant changes than male students do in the majority of risk behaviours.

The disparity in the reduction of risk behaviours among provinces is seen in Table 7. For example, provinces 2, 5, and 7 did better in improving the self-reported CVD risk behaviours compared to that of in other provinces. This could be due to reduced disaggregated sample size of students by province.

Although a statistically significant reduction was noted in the eating of junk food and consumption of drinks and an increase in the consumption of healthy food like boiled egg, peanuts and maize, as seen in Table 4, the percentage changes in the consumption of other varieties of healthy food such as beaten rice, mixed fruits and vegetables was not found at a statistically significant (P > 0.05) level. This may be due to various factors such as children being compelled to eat junk food because of a lack of other options, as pointed out by the 2015 BISHES baseline study. Parents, especially in public schools, were not sending healthy snacks mainly because of lack of time. Similarly, financial constraints could be another factor, as suggested by similar research works that the predictors of food choices include income, urbanization, and marketing strategy of the food industry, as well as consumers’ choices and preferences [31].

Nevertheless, highly motivated participation of school teachers in the study, along with their felt positive experience about the programme’s impact could be an important predictor for sustainability of such school-based interventions [32]. The KIs echo similarly. They viewed that it was feasible in Nepal to embedding the experiential learning modules in the existing contents of the NCD school curriculum with no added costs to the schools. In this regard, teachers could be a hub for creating a snowball effect for changing risk behaviour in school communities.

A few strategically important implications can be drawn from the study. First, as the BAT integrative intervention with experiential learning approach has been able to bring about significant positive change in self-reported CVD risk behaviours among school adolescents after the intervention, its accreditation by the Government of Nepal stands strong. Second, the integrated experiential learning modules were popular among the students and teachers alike in transferring knowledge from direct experience in a playful manner. This is not only an important mileage in and of itself, but an important hub for creating a snowball effect for changing risk behaviour in school communities. Third, the indirect social effect generated during the robust interactions among all the concerned stakeholders at every phase of the BAT intervention cannot be undermined as an important factor associated to improved CVD behaviour change. Finally, as echoed by school teachers, it seems possible to embed the BAT integrated learning modules in the existing NCD school curriculum at no additional cost.

However, some caution should be exercised in interpreting the results presented here. First, a placebo-control group was not used in this study, which is one of the notable limitations of the study. Second, because the focus of the study was on assessing to what extent the experiential learning intervention modules could influence the five CVD risk behaviours in aggregate based on self-reported responses of the students, thus, it is subject to information bias.

Third also that the survey responses were anonymous at both pretest and post-test, we couldn’t measure intra-student behaviour change after the intervention, and only the aggregate changes based on students’ self-reported responses in behaviours are presented here.

Similarly, we did not take into account the social determinants of health, such as family income, parent’s education as well as other community amenities, which could be potential factors, among others, affecting students’ behaviour change.

## Conclusion

In conclusion, the sample in this study has shown positive changes in school adolescents’ self-reported aggregate CVD risk behaviours. These changes occurred after a only 25 credit hours of intervention using the BAT experiential learning modules. However, positive health outcomes are only possible when such risk behaviour changes are sustained and scaled. To this effect, a further study may be in order to explore sustainability and scaling of these learning modules through the existing non-communicable disease (NCD) school curriculum activities in Nepal.
